# A dominant *dpy-10* co-transformation marker using CRISPR/Cas9 and a linear repair template in *Caenorhabditis tropicalis*

**DOI:** 10.17912/micropub.biology.000900

**Published:** 2023-11-03

**Authors:** Montana Bobinski, David Pilgrim

**Affiliations:** 1 Department of Biological Sciences, University of Alberta, Edmonton, Alberta, Canada

## Abstract

*Caenorhabditis* *elegans* is an excellent genetic model system with a large arsenal of forward and reverse genetic techniques. However, not all approaches are easily ported to related *Caenorhabditis* species (which are useful for gene conservation and gene pathway evolution studies). For CRISPR/Cas9 genetic editing, an easily screenable and dominant co-transformation marker is required – a secondary mutation that won’t impact the phenotype of a desired mutation but is capable of being screened for in heterozygous mutants. We describe here the adaptation of a dominant dumpy/roller CRISPR/Cas9-induced mutation in the *C. tropicalis* *dpy-10* orthologue.

**Figure 1. Identification of the C. tropicalis dpy-10 orthologue and generation of a dominant dpy-10 mutation:**
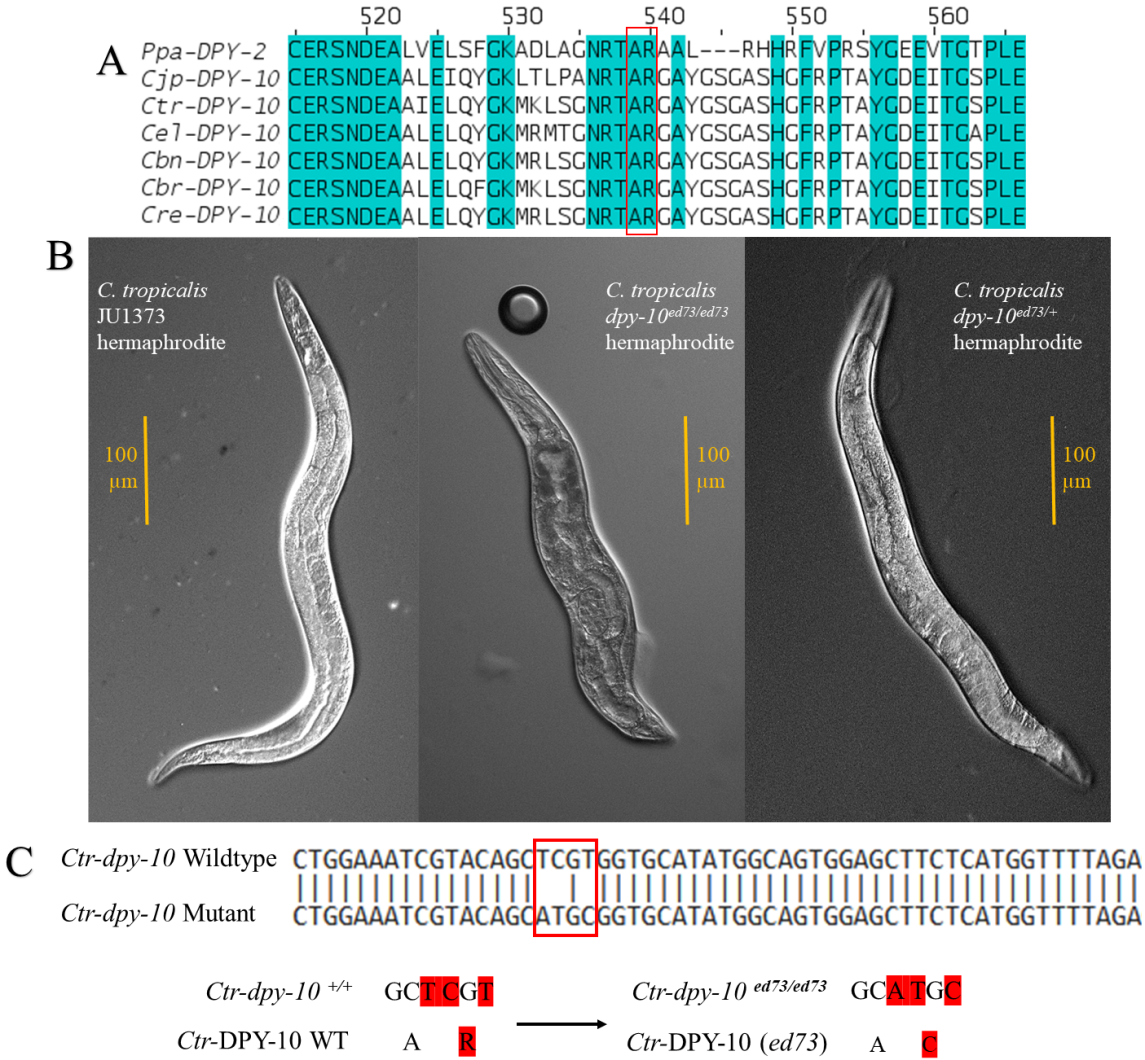
(A) Jalview-visualized amino acid sequence alignment between *P. pacificus* (Ppa) *, C. japonica* (Cjp), *C. tropicalis* (Ctr), *C. elegans* (Cel), *C. brenneri* (Cbn), *C. briggsae* (Cbr), and *C. remanei* (Cre) DPY-10 orthologues. Protein alignments were generated using CLUSTAL OMEGA at default settings, and teal bands indicate 100% amino acid identity across all sequences and species. The red box highlights the alanine(A)→arginine(R) sequence that was mutated by Paix *et al.* in *C. elegans* to generate the *Cel-dpy-10* dominant mutation. (B) Differential interference contrast (DIC) microscopy images at 400x resolution (40x objective, 10x ocular) of a wildtype *C. tropicalis* JU1373 adult hermaphrodite (left), a *C. tropicalis dpy-10(ed73)* adult homozygous mutant hermaphrodite (middle), and a *C. tropicalis dpy-10(ed73)* adult heterozygous roller hermaphrodite (right). Note that the *Ctr-dpy-10(ed73)* homozygous mutant worm is noticeably shorter and fatter than the wildtype hermaphrodite, while the roller is nearly indistinguishable to the wildtype *C. tropicalis* worm. 100 μm scale bar is included. (C) *Ctr-dpy-10* wildtype and *Ctr-dpy-10* ( *ed73)* DNA sequence alignment (top), indicating the TCGT>ATGC mutation in the *Ctr-dpy-10* ( *ed73)* worms. This mutation causes the arginine amino acid coded by the CGT codon to be substituted (black arrow) by a cysteine amino acid coded by the TGC codon.

## Description

Developing genomic editing tools in the non-model system *Caenorhabditis tropicalis* .

The availability of genome sequences from a wide variety of species has allowed the field of evolutionary developmental biology to thrive. However, while the well-established model metazoans such as *Drosophila melanogaster* and *Caenorhabditis elegans* have a wealth of genetic and molecular tools at their disposal for transgenesis, cell or gene product tagging, or the targeted alteration of gene expression and function, most species do not. In these cases, there are limited options available for developing gene editing pipelines, due to the lack of robust techniques and controls.

The androdioecious nematode *Caenorhabditis tropicalis* is one such example. A relative of *C. elegans* , it is of particular interest for the study of traits such as population genetic diversity, selfish elements, and the evolution of self-fertility from strictly outcrossing ancestors (Noble et al. 2020; Ben-David et al. 2021; Andersen & Rockman 2022) . Like *C. elegans* , *C. tropicalis* is easy and inexpensive to work with and maintain in the lab, possesses a transparent body with easily identifiable internal structures, is self-fertile with a short generation cycle of approximately 3.5 days, and has a sequenced genome (Stein et al. 2003; Meneely et al. 2019; Noble et al. 2020) .

However, neither genetic mutations nor transgenic lines have yet been reported for *C. tropicalis* . Here we report the adaptation of a tool from *C. elegans* where we use CRISPR/Cas9 to alter the *C. tropicalis* orthologue of *dpy-10* into a dominant marker, to improve the identification of successful gene editing of unlinked recessive targets.

*Dpy-10* as a co-transformation marker for CRISPR/Cas9 use in *Caenorhabditis* .

Injections of CRISPR/Cas9 reagents in the gonadal arms generally lead to genetic edits in haploid oocytes, which are then fertilized by non-edited haploid sperm to produce heterozygous, diploid zygotes (Kim et al. 2022) . As most mutations are recessive in their phenotype, for CRISPR/Cas9 mutants to be effectively screened for in *C. tropicalis* , we required a gene that can be co-transformed in a manner that produces a dominant phenotype, so that heterozygous F1 mutants can be easily screened for amongst their wildtype siblings (Nanjundiah 1993; Kim et al. 2022) . One such gene, in *C. elegans* and *C. briggsae* , is *dpy-10* (Kim et al. 2022) .

For hermaphroditic *Caenorhabditis* worms, *dpy-10* can be used as a dominant co-transformation marker for CRISPR/Cas9, as the dumpy and/or roller phenotype does not affect their viability or fertility (Levy et al. 1993; El Mouridi et al. 2017; Hung et al. 2017; Paix et al. 2017; Smith et al. 2020) . Notably, a single four-base pair edit in the *C. elegans dpy-10* gene can produce a dominant mutation, where the heterozygotes roll along the long axis of their body - a roller phenotype - and where the homozygotes appear shorter and fatter than roller or wildtype worms - a dumpy phenotype (Arribere et al. 2014; Paix et al. 2017). We wanted to test whether the *C. tropicalis dpy-10* orthologue could similarly be edited as a dominant co-transformation marker.

Identification of the *C. tropicalis dpy-10* orthologue.

TBLASTN identified the *Ctr-dpy-10* orthologue, and reciprocal BLASTP identified *C. elegans* and *C. briggsae* DPY-10 as the strongest matches (Altschul et al. 1990; Moreno-Hagelsieb and Latimer 2008; Davis et al. 2022) . CLUSTAL OMEGA and Jalview were then utilized for the generation and visualization of, respectively, pairwise predicted protein sequence alignments between the DPY-10 orthologues across multiple *Caenorhabditis* species and *Pristionchus pacificus* (Waterhouse et al. 2009; Goujon et al. 2010; Sievers et al. 2011) . The specific arginine residue [Arg92 in the *C. elegans* protein (Levy et al. 1993) ] , where CRISPR/Cas9 editing to cysteine results in a dominant phenotype in *C. elegans* (Paix et al. 2017) , was discovered to be perfectly conserved across all *Caenorhabditis* and *Pristionchus* species ( Figure 1A ).

Precision gene editing in *C. tropicalis* using CRISPR/Cas9 and a ssODN repair template.

Based on the success of the *C. elegans dpy-10* co-transformation marker, our CRISPR/Cas9 protocol for producing the specific edits necessary to generate the dominant *Ctr-dpy-10* mutation required only the inclusion of single-stranded oligodeoxynucleotides (ssODNs) as a repair template (Paix et al. 2017; Ghanta et al. 2021) . This also shows the adaptability of a *C. elegans* HDR CRISPR/Cas9 protocol in related nematode species (Lo et al. 2013) .

Five days after the injection of 18 *C. tropicalis* JU1373 young adult hermaphrodites using the CRISPR/Cas9 editing mix, from 3 of the injected P0 worms, 12 out of 67 total adult F1 offspring presented a roller phenotype, which indicates an editing efficiency similar to the previously reported 16-18% positive HDR CRISPR/Cas9 rates in *C. elegans* zygotes (Li et al. 2022) . With each rolling F1 offspring, approximately one-quarter of their F2 offspring presented a dumpy phenotype, consistent with homozygosity for the *Ctr-dpy-10* mutation ( Figure 1BC ). F2 dumpy worms then underwent clonal expansion on individual plates, in which all F3 offspring were observed to likewise be dumpy. PCR amplification and sequencing indicated that all lysed specimens conformed with the designed TCGT>ATGC *Ctr-dpy-10* CRISPR edit (designated *ed73* ), which had the predicted effect of substituting the arginine codon CGT to the cysteine codon TGC, producing a dominant dumpy phenotype ( Figure 1C ).

## Methods

*C. tropicalis* culturing and maintenance.

*C. tropicalis* wildtype JU1373 (Kiontke et al. 2011) was obtained from the *Caenorhabditis* Genetics Center (CGC) at the University of Minnesota. *C. tropicalis* hermaphrodites were cultured on NGM agar plates streaked with *E. coli* OP50 , as described for *C. elegans* (Stiernagle 2006) . The *C. tropicalis* DP485 *dpy-10(­ ed73 )* strain has been archived at the CGC.

Primer Sequences.

Predicted *Ctr-dpy-10* protein-coding sequences were aligned using BLASTN as the “Query Sequence” to the wildtype version of *Cel- dpy-10* ssODN (change ATGC to TCGT), which acted as the “Search Set”, to identify the specific region to target (Paix et al. 2017) . The Caenorhabditis_sp11_ JU1373 -3.0.1 GCA_000186765.1 genome scaffold assembly was submitted to CRISPOR, after which the identified *Ctr-dpy-10* region was analyzed. The guide sequence was selected based on three criteria: high “MIT Specificity Score”, high “CFD Spec. Score”, and PAM sequence being within 5 bp of the intended edit site. CRISPOR was used to help design and evaluate the on/off target potential of guide sequences for *C. tropicalis* CRISPR/Cas9 (Concordet and Haeussler 2018) . The Alt-R CRISPR HDR Design Tool and PrimerQuest™ programs (IDT) were used to design the guide RNA (CRISPR RNA; crRNA) and ssODN linear repair template (Concordet and Haeussler 2018) .

*Ctr-dpy-10* primers (below) were used to amplify the predicted CRISPR-edited region for sequencing.

***Ctr-dpy-10* Guide Sequence:** CGTACAGCTCGTGGTGCATA

***Ctr-dpy-10* ssODN:** TTCAATACGGAAAAATGAAACTTTCTGGAAATCGTACAGCATGCGGTGCATATGGCAGTGGAGCTTCTCATGGTTTTAGACCAACTGCT

***Ctr-dpy-10* Forward Primer:** AACTATTCGCGTCAGATGAT

***Ctr-dpy-10* Reverse Primer:** CTCCATAAGCAGTTGGTCTA

Identification of the *C. tropicalis dpy-10* orthologue.

DPY-10 predicted protein sequences for *C. elegans* and *C. briggsae* , collected from WormBase, were used as the “Query Sequence” for TBLASTN, and a Whole-genome Shotgun Contigs (wgs) database for *Caenorhabditis tropicalis* was used as the “Search Set”. No algorithm parameters were altered. The *C. tropicalis* JU1373 accession set AEKS01003488.1: Contig629.1541 was identified as the container for the *Ctr-dpy-10* orthologue and was chosen based on possessing the following elements: highest “Max Score”, “Total Score”, and “Query Cover”; and lowest “E-value” (Altschul et al. 1990; Davis et al. 2022) . The accompanying *C. tropicalis* NIC203 accession set was used to determine that the *Ctr-dpy-10* gene is syntenic with the *C. elegans* and *C. briggsae* *dpy-10* orthologues.

A reciprocal best hit strategy was also implemented: the CTR-DPY-10 predicted protein sequence, collected from WormBase-Compara, was used as the “Query Sequence” for BLASTP, and a “Non-Redundant Protein Sequences (nr)” database for *C. elegans* or *C. briggsae* was used as the “Search Set”. No algorithm parameters were altered.

Homology-directed repair CRISPR/Cas9 in *C. tropicalis* .

Please refer to Ghanta et al. 2021 for CRISPR reagent preparation, injection of young adult *C. tropicalis* hermaphrodites, worm recovery, and handling. Editing efficiency was calculated as the percentage of injected worms which produced F1 rollers and the proportion of F1 progeny which displayed the roller phenotype.

Individual injected P0 *C. tropicalis* hermaphrodites were separated onto individual plates and the F1 offspring were screened over 3-5 days for any presenting a roller phenotype. No F1 dumpy worms were observed. These F1 rollers were then separated onto individual plates and their offspring were screened over 3 days to identify F2 dumpy worms. These F2 dumpy worms were likewise separated onto individual plates and allowed to undergo clonal expansion.

PCR amplification of *Ctr-dpy-10* .

Lysis protocol was adapted from Dr. Ian Chin-Sang. Worms were washed from a single NGM plate and resuspended in 100 µL of lysis solution (1X PCR buffer with 1.5 mM MgCl _2_ and 1 mg/mL Proteinase K). Tubes containing lysis solution and worms were spun down and cooled in liquid nitrogen for 10 minutes before being heated to 65°C for 2 hours, followed by 95°C for 30 min. The resulting *C. tropicalis* genomic DNA solution can be diluted 100-fold and used as a PCR template.

The *Ctr-dpy-10* forward and reverse primer sequences are reported above. The thermocycler protocol is: 1 x 3’ 94°C; 35 x (45s 94°C, 30s 52.5°C, 90s 72°C); 1 x 10’ 72°C. *Ctr-dpy-10* PCR products were purified using a QIAquick® PCR Purification Kit, and purified DNA was quantified using a NanoDrop® ND-1000 UV-Vis spectrophotometer. Sanger sequencing was performed by the MBSU at the University of Alberta.

## Reagents

**Table:** 

**Gene**	**Strain**	**Available From**	**Source**
*C. tropicalis* Ancestral Wildtype	JU1373	CGC	Kiontke et al. 2011; *Caenorhabditis* Genetics Center (CGC)
* Ctr-dpy-10( ed73 ) *	DP485	CGC	This work
